# Apigenin Inhibits IL-31 Cytokine in Human Mast Cell and Mouse Skin Tissues

**DOI:** 10.3390/molecules24071290

**Published:** 2019-04-02

**Authors:** Denis Nchang Che, Byoung Ok Cho, Jae Young Shin, Hyun Ju Kang, Ji-Su Kim, Hyeonhwa Oh, Young-Soo Kim, Seon Il Jang

**Affiliations:** 1 Department of Food Science and Technology, Chonbuk National University, Jeonju-si, Jeollabuk-do 54896, Korea; chedenis88@gmail.com (D.N.C.); ohw2825@naver.com (H.O.); 2 Department of Health Management, Jeonju University, Jeonju-si, Jeollabuk-do 55069, Korea; enzyme21@naver.com (B.O.C.); kim1011003@naver.com (J.-S.K.); 3 Research Institute, Ato Q&A Co., Ltd., Jeonju-si, Jeollabuk-do 54840, Korea; sjy8976@naver.com (J.Y.S.); dkgk0608@naver.com (H.J.K.)

**Keywords:** IL-31, apigenin, mast cells, atopic dermatitis, MAPK, NF-κB

## Abstract

IL-31 is a recently discovered cytokine that is produced not only in T-cells but also in mast cells. It is strongly implicated to play a key role in inflammatory diseases and in the pathogenesis of itch in atopic dermatitis. Apigenin, a flavonoid of plant origin has numerous biological applications. In this study, we showed that apigenin modulates IL-31 mRNA, protein expression, and release in stimulated human mast (HMC-1) by inhibiting the phosphorylation activation of MAPK and NF-κB. To determine whether apigenin has similar effects in vivo, using Compound 48/80, we developed an atopic dermatitis itch model in mice and found an increase in IL-31 expression in the skin. We also revealed that apigenin prevents the infiltration and degranulation of mast cells and suppressed mRNA and protein expression of IL-31 in the skin of mice. These results provide a new suggestion of the potential applicability of apigenin for treatment of various inflammatory diseases and itch mediated by IL-31.

## 1. Introduction

IL-31 is a recently discovered helical inflammatory cytokine that is derived from the gp130/IL-6 cytokine family, which consists of other cytokines such as IL-6 [1]. Its mRNA was first discovered in activated CD4^+^ T cells [1]. Recently, the mRNA has also been found in activated mast cells [2]. IL-31 signals through IL-31 receptor alpha (IL-31RA) that is coupled with an oncostatin M receptor beta (OSMR). The receptors are constitutively expressed in testis, bone marrow, skeletal muscle, kidney, colon, thymus, small intestine, trachea, and dorsal root ganglia and in activated monocytes [1]. IL-31 is known to play a key role in inflammatory diseases. Zhang et al. (2008) found that IL-31 levels were elevated in skin biopsies of patients with atopic dermatitis compared to patients without atopic dermatitis [3]. The study found that IL-31 induced CCL1, CCL17, and CCL22 chemokines in atopic dermatitis-irritated skin. In addition, IL-31 was also found to play a significant role in pruritus (itch) in atopic dermatitis. For example, IL-31 mRNA was expressed in a mouse model of atopic dermatitis experiencing pruritus than in mice model of atopic dermatitis not experiencing pruritus [4]. Hawro et al. (2014) [5] reported the late onset of IL-31-induced itch in a human study. Interestingly, the IL-31 receptor complex is abundant in the dorsal root ganglia where itch sensation is thought to originate in human tissues [3]. Aside from the role of IL-31 in atopic dermatitis, it is believed that IL-31 plays a role in inflammatory bowel disease and airway hypersensitivity [6,7].

Therefore, suppressing the expression and production of IL-31 in cellular processes will be beneficial for the treatment of chronic inflammatory diseases, including but not limited to atopic dermatitis, pruritus, inflammatory bowel diseases, and airway hypersensitivity. While several immunomodulatory agents are used to treat chronic inflammatory diseases, this often comes with serious side effects. Moreover, other agents have been proven ineffective for treating itch in atopic dermatitis. For instance, the antihistamine drug used to treat pruritus in atopic dermatitis fails, as histamine is not the only mediator of pruritus in atopic dermatitis [8]. Long-term use of nonsteroidal anti-inflammatory drugs and corticosteroids used for treating chronic inflammation are linked to an increased risk of several conditions, including skin atrophy, stretch marks, muscle weakness, vision problems, cardiovascular disease, cerebrovascular disease, high blood pressure, osteoporosis, peptic ulcer, and kidney diseases [9,10]. Therefore, the search for other alternative therapies for the treatment of chronic inflammatory diseases including itch in atopic dermatitis cannot be overlooked.

Apigenin, a naturally occurring flavonoid of plant origin, abundantly present in common vegetables, including celery, parsley, and onions, has remarkable biological applications. [11]. Apigenin has been shown to reduce IL-6, IL-1β, TNFα, COX-2, and NO release in lipopolysaccharide-stimulated macrophages and lipopolysaccharide-induced lung inflammation [12,13]. Apigenin causes cell cycle arrest in leukemia cells and induces autophagy, thereby supporting a potential chemopreventive role [14]. Evidence of its biological activities has also been exemplified in animal studies that show that apigenin readily crosses the blood–brain barrier with no toxicity at high doses and can prevent neuroinflammation, associated with Alzheimer’s disease [15]. These biological properties of apigenin are mediated by its down-modulation of the constitutive expression of NF-κB/p65 [16]. However, the ability of apigenin to modulate the newly discovered IL-31 cytokine responsible for itch in atopic dermatitis has not been investigated.

Therefore, the objective of this study was to investigate the effect/mechanism of the action of apigenin in modulating IL-31 release in human mast cells (HMC-1) and in compound 48/80-induced itch in atopic dermatitis. Mast cells are one of the major effector cells in the immune response system and secrete pro-inflammatory cytokines including IL-31 [17]. Here, we hypothesized that apigenin treatment can suppress the release of IL-31 in phorbol myristate acetate and ionomycin (PI)-stimulated HMC-1 cells and compound 48/80-induced itch in mice.

## 2. Results

### 2.1. Effects of Apigenin on Cell Viability of HMC-1 Cells

**Figure 1:**
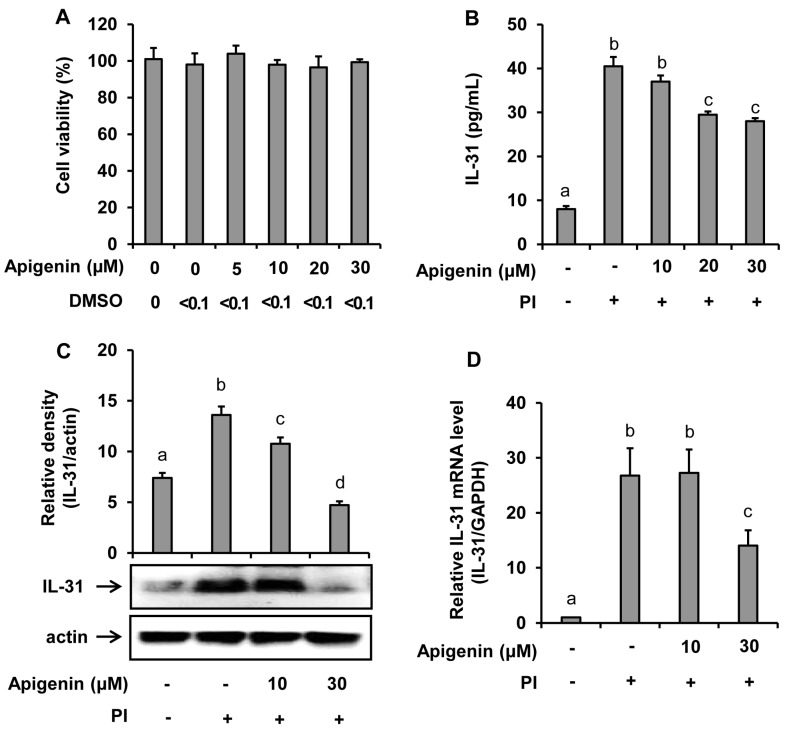
Effects of apigenin on the human mast cells (HMC-1) viability and IL-31 production by HMC-1. Cells were pre-treated with apigenin at indicated concentrations for 24 h. Cell viability was determined by EZ-Cytox assay (**A**). Cells were pre-treated with apigenin at indicated concentrations for 1 h prior to PI stimulation for 12 h (**B**), 6 h (**C**), and 3 h (**D**). ELISA was used to measure IL-31 released in the cell culture media (**B**). Western blot analysis was used to determine the IL-31 protein expression levels in the cells and the bands were quantitated using Image J analysis software tool (**C**). Reverse-transcription (RT)-PCR was performed to determine the level of expression of IL-31 mRNA in the cells (**D**). The values presented are the means ± SD of three independent experiments. Bars with different small case letters indicated statistically significant difference at *p* < 0.05. PI, phorbol-12-myristate 13-acetate (50 nM) and ionomycin (1 μM).

Water-soluble tetrazolium (WST) cell viability assay was used to test whether apigenin had a cytotoxicity effect on HMC-1 cells. As shown in Figure 1A, apigenin at concentrations of up to 30 μM showed no cytotoxicity on HMC-1 cells. In addition, treatment with dimethyl sulfoxide (solvent used to dissolve apigenin for this study) at a concentration of 0.1% showed no cytotoxicity on HMC-1 cells.

### 2.2. Effects of Apigenin on PI-Induced IL-31 Release in HMC-1 Cells

The suppressive effect of apigenin in the IL-31 mRNA expression and IL-31 protein production and release into the cell culture media was investigated. The results as revealed in Figure 1 showed that PI significantly stimulated the mRNA expression in addition to protein production and release into the culture medium. However, when the HMC-1 cells were treated with apigenin at either 10 µM, 20 µM, or 30 µM concentrations before stimulated with PI at indicated time intervals, a significant decrease of IL-31 release into the culture medium was observed with apigenin 20 µM and 30 µM treatments (Figure 1B). A significant decrease of IL-31 protein and IL-31 mRNA expression at 30-µM apigenin treatment were also observed (Figure 1C,D).

### 2.3. Effects of Apigenin on MAPK Cascade Signaling Pathways in Stimulated HMC-1 Cells

**Figure 2:**
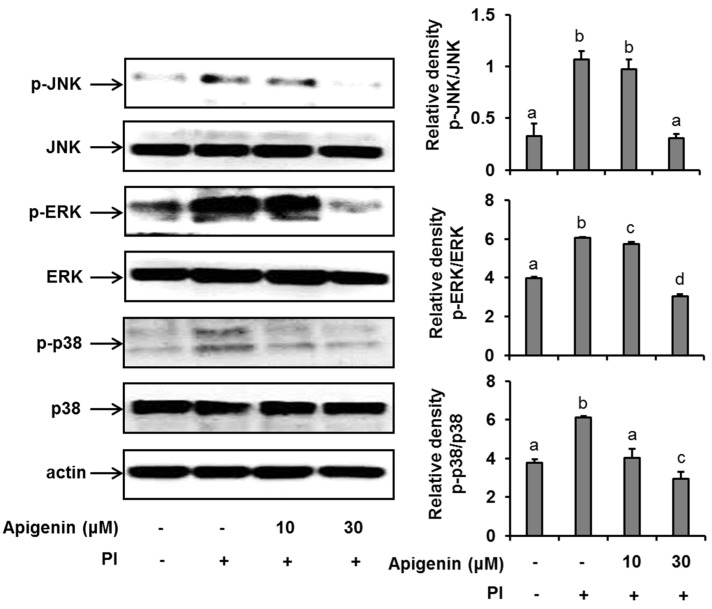
Effects of apigenin on MAPK cascade signaling pathways in HMC-1 cells. Cells were pre-treated with apigenin at indicated concentrations for 1 h prior to PI stimulation for 30 min. Western blot analysis was performed to evaluate the expressions of molecules of the MAPK cascade signaling pathways in the cells. The bands were quantitated using Image J analysis software tool. The data presented are the means ± SD of three independent experiments. Bars with different small case letters indicated statistically significant difference at *p* < 0.05. PI, phorbol-12-myristate 13-acetate (50 nM) and ionomycin (1 μM).

Since apigenin had shown to inhibit the expression and release of IL-31 in PI-stimulated cells, we sought to explore the mechanisms by which apigenin inhibits IL-31 in HMC-1 cells. As shown in Figure 2, when HMC-1 cells were stimulated with PI for 30 min, we found that the phosphorylation of ERK, JNK, and p38 was significantly enhanced. However, when the HMC-1 cells were pre-treated with 10 µM and 30 µM of apigenin for 1 h before stimulation with PI, there was a significant suppression of ERK, JNK, and p38 phosphorylation, thus indicating an inhibition in their activations.

### 2.4. Effects of Apigenin on NF-κB Pathway Activation in Stimulated HMC-1 Cells

**Figure 3:**
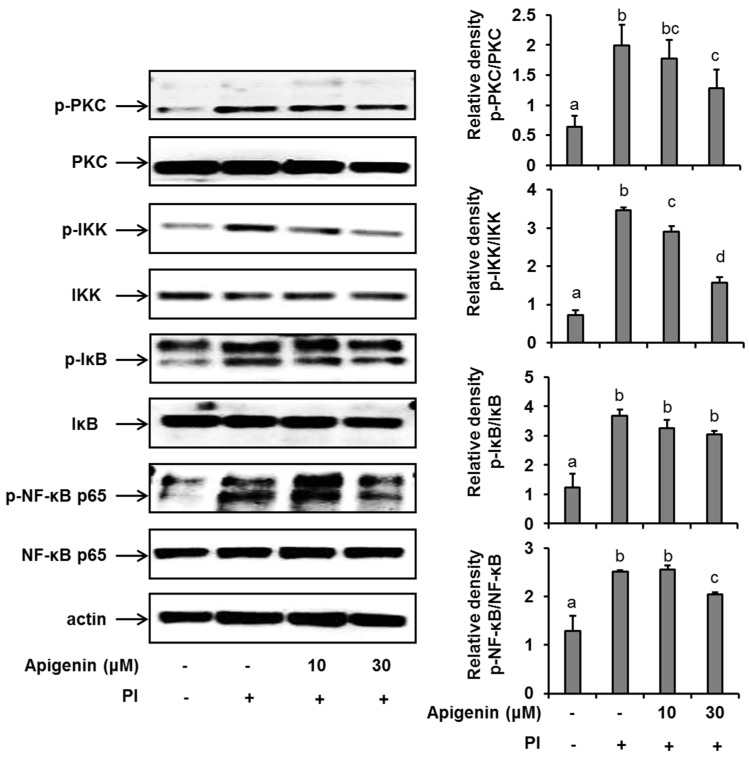
Effects of apigenin on the NF-κB signaling pathway in HMC-1 cells. Cells were pre-treated with apigenin at indicated concentrations for 1 h prior to PI stimulation for 30 min. Western blot analysis was performed to evaluate the expressions of molecules of the NF-κB signaling pathway in the cells. The bands were quantitated using Image J analysis software tool. The data presented are the means ± SD of three independent experiments. Bars with different small case letters indicated statistically significant difference at *p* < 0.05. PI, phorbol-12-myristate 13-acetate (50 nM) and ionomycin (1 μM).

In an effort to further explore the mechanism of action of apigenin in suppressing IL-31 expression and release, we investigated the effects of apigenin on a series of kinases that are involved in the NF-κB pathway activation in PI-stimulated HMC-1 cells. We found that following the stimulation of HMC-1 cells with PI for 30 min, there was a significant up-regulation (compared to unstimulated cells) of the phosphorylation of PKC, IKKβ, and IκBα and finally of the phosphorylation of Ser536 of the p65 subunit of the transcription factor, NF-κB in HMC-1 cells (Figure 3). Moreover, when HMC-1 cells were pre-treated with apigenin at a concentration of 30 µM for 1 h before PI stimulation, we recorded a significant suppression of the phosphorylation of all these kinases, including NF-κB’s p65 subunit. Considering all these results, we concluded that apigenin treatment regulated PI-induced MAPK and NF-κB/p65 signaling in HMC-1 cells, thus regulating IL-31 release in HMC-1 cells. We had previously shown that chemical inhibitors of ERK, JNK, and p38 and the NF-κB transcription factor prevented the production of IL-31 in HMC-1 cells [18].

### 2.5. Effects of Apigenin on Compound 48/80-Induced Itch in Mice Skin

**Figure 4:**
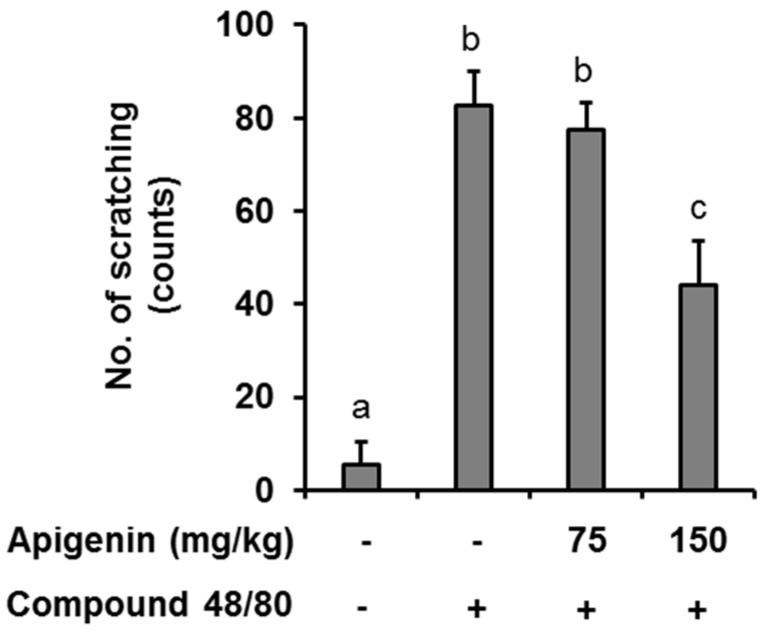
Effects of apigenin on compound 48/80 itch in mice. Five mice each were placed into four groups: Compound 48/80 negative control; compound 48/80 positive control; compound 48/80 with apigenin 75 mg/kg treatment; and compound 48/80 with apigenin 150 mg/kg treatment. Compound 48/80 (50 μg/site, 0.1 mL each) was injected into the dorsal dermis of the mice neck 60 min after administration of 2% geletin or apigenin at indicated concentrations. The number of scratches were monitored with micro-cameras for 30 min and counted in a double-blinded manner. Data are presented as the mean ± SEM of five mice per group. Bars with different small case letters indicated statistically significant difference at *p* < 0.05.

In an in vivo case study, we evaluated the anti-itch effect of apigenin in an atopic dermatitis itch model induced by compound 48/80. It was found that injection of compound 48/80 (50 µg) into the dorsal dermis of the mouse resulted in an increase in scratching behavior while no scratching behavior was observed in normal mice. However, oral apigenin treatment at 150 mg/kg significantly prevented itch in the mice (Figure 4).

### 2.6. Effects of Apigenin on Compound 48/80-Induced Infiltration of Polymorphic Nuclear Leukocyte in Mice Skin

**Figure 5:**
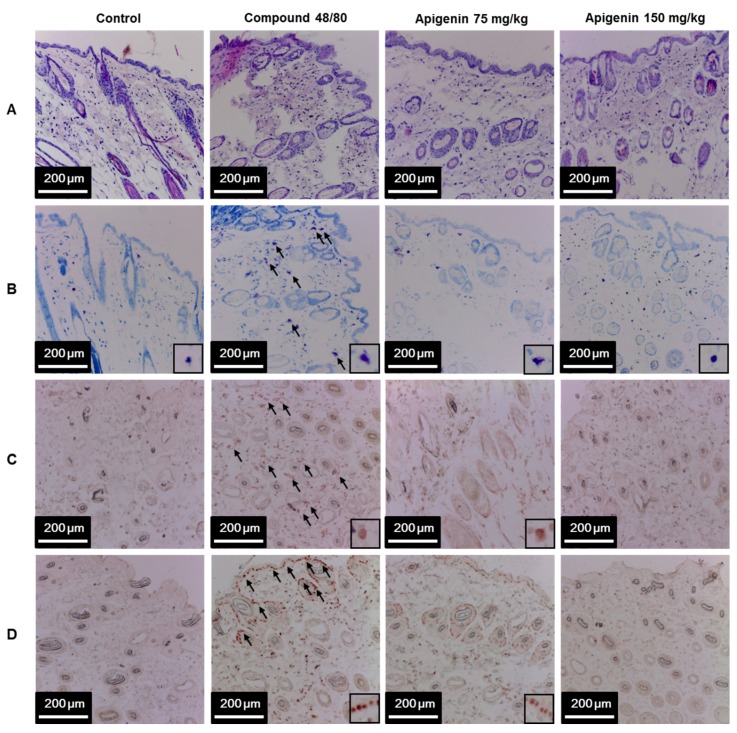
Effects of apigenin on compound 48/80-induced infiltration of mast cell and IL-31 expression in mice skin. Five mice each were placed into four groups: Compound 48/80 negative control; compound 48/80 positive control; compound 48/80 with apigenin 75 mg/kg treatment; and compound 48/80 with apigenin 150 mg/kg treatment. Compound 48/80 (50 μg/site, 0.1 mL each) was injected in the dorsal dermis of the mice neck 60 min after administration of 2% gelatin or apigenin at indicated concentrations. Skin tissue sections were stained with hematoxylin/eosin stains showing infiltration of polymorphonuclear leukocytes (**A**); toluidine blue showing mast cell infiltration into the skin (**B**); immunohistochemistry was done to demonstrate tryptase (**C**) and IL-31 (**D**) in the mice skin.

**Figure 6:**
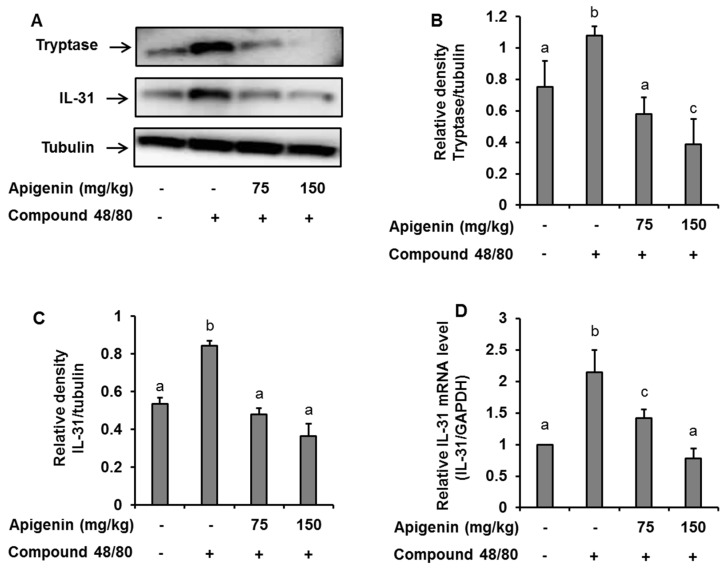
Western blot and RT-PCR analysis showing the effects of apigenin on compound 48/80-induced tryptase and IL-31 expression in the mice skin. Five mice each were placed into four groups: Compound 48/80 negative control; compound 48/80 positive control; compound 48/80 with apigenin 75 mg/kg treatment; and compound 48/80 with apigenin 150 mg/kg treatment. Compound 48/80 (50 μg/site, 0.1 mL each) was injected in the dorsal dermis of the mice neck 60 min after administration of 2% gelatin or apigenin at indicated concentrations. (**A**) Western blot analysis of tryptase and IL-31 protein expression. The bands were quantitated using Image J analysis software tool (**B**,**C**). (**D**) RT-PCR showing the expression of IL-31 mRNA in the mice skin. The data presented are the means ± SD of five mice per group. Bars with different small case letters indicated statistically significant difference at *p* < 0.05.

To assess the effects of apigenin on scratching behavior, we sectioned skin tissues around the neck of the mice at the site of compound 48/80 injection and prepared paraffin sections with hematoxylin and eosin to observe the infiltration pattern of polymorphic nuclear leukocytes (PMNL). We found that compound 48/80 induced the infiltration of PMNL into the mice skin while the group treated with apigenin showed lesser infiltration of the PMNL (Figure 5A). Again, we stained the tissues with toluidine blue to observe mast cells infiltration into the skin tissues. Interestingly, compound 48/80 injection also led to the infiltration of mast cells into the mice skin, while the apigenin-treated groups showed less infiltration of the mast cells (Figure 5B). To investigate mast cell activation and the effects of apigenin on mast cell activation, we evaluated the expression of tryptase in the mice skin using western blot and immunohistochemistry analysis methods. As expected, compound 48/80 led to an increase in the expression of tryptase in the mice skin while treatment with apigenin dose-dependently prevented the expression of the tryptase (Figure 5C and Figure 6A,B).

### 2.7. Effects of Apigenin on Compound 48/80-Induced Expression of IL-31 in Mice Skin

Since IL-31 is strongly associated with itch in atopic dermatitis, we investigated the effects of apigenin on compound 48/80-induced expression of IL-31 mRNA and protein in the dorsal skin of the mice. Through quantitative RT-PCR, western blot, and immunohistochemistry, we found that injection of compound 48/80 led to increase in the expressions of IL-31 mRNA and its subsequent protein expression in the skin of the mice. On the other hand, treatment with apigenin resulted in the suppression of IL-31 mRNA and protein expression (Figure 5D and Figure 6A,C,D).

## 3. Discussion

IL-31 has been implicated in several inflammatory diseases, including atopic dermatitis, pruritus (especially in atopic dermatitis), inflammatory bowel diseases, and skin and airway hypersensitivity. Therefore, the suppression of IL-31 in cellular and tissue processes is deemed important for the prevention and treatment of such diseases. Researchers believe that identifying and eliminating and/or suppressing the triggering elements that mediate itch in atopic dermatitis will be crucial for managing itch in atopic dermatitis [19]. In this light, we sought to investigate whether apigenin could suppress the expression and release of the newly discovered inflammatory and itch-related IL-31 cytokine in HMC-1 cells and to determine if these effects could be translated to an in vivo setting.

In the present study, our preliminary experiments found a first significant increase in the release of IL-31 cytokine in the cell medium, expressions of the IL-31 protein in the cell, and the expression of IL-31 mRNA 12 h, 6 h, and 3 h, respectively, after stimulation of HMC-1. The time difference is because these processes occur in the cells at different times. Usually, it takes a couple of minutes for the signaling pathway to be activated so that transcription factors can bind to DNA to initiate transcription. After transcription, it also takes some time for the mRNA formed from the transcription process to be processed, to be exported to the cytosol, for translation to be initiated, and for translation elongation to occur. Each mRNA must then be translated multiple times until enough molecules of the protein can be detected and subsequently released [20,21]. As a result, we applied the time of the first significant release or expression of Il-31 protein and IL-31 mRNA expression in our study. According to the study, apigenin, which had no cytotoxicity to HMC-1 cells up to 30 μM, prevented the release of IL-31 cytokine in HMC-1 cells stimulated by PI. Further investigation also revealed that apigenin also suppressed the expression of the IL-31 protein and IL-31mRNA in such stimulated cells thus implying that the action of apigenin in suppressing IL-31 release could potentially begin by regulating the expression of IL-31 mRNA in HMC-1 cells. Previous studies revealed that apigenin decreased the mRNA expressions of other cytokines such as IL-6, TNFα, IL-1β, and IL-8 in LPS, PI, or di-(2-ethylhexyl) phthalate-stimulated human umbilical vein endothelial cells, human monocytes, and/or mouse macrophages [22,23,24]. Thus, apigenin can be further investigated as a promising agent for preventing and treating various inflammatory diseases including those specifically mediated by IL-31.

MAPK cascade-signaling pathways play an essential role in the regulation of the expression of cytokines. The activation of these pathways through PKC’s activation by PI results in the activation of various genes including inflammatory cytokine genes such as IL-1β, IL-6, and TNFα [25,26]. This makes MAPK a major target for the treatment of inflammatory and allergic diseases. To understand the mechanism of interaction of apigenin in suppressing the expression of IL-31 mRNA and hence its protein expression and the cytokine released in HMC-1 cells, we investigated the phosphorylation status of ERK, JNK, and p38 in the MAPK/ERK, MAPK/JNK, and MAPK/p38 signaling pathways in the HMC-1 cells. Preliminary studies showed that all the MAPK signaling molecules were first significantly expressed 30 min after the HMC-1 cells were stimulated. Subsequent studies at 30 min after the stimulation of HMC-1 showed that apigenin did suppress the phosphorylation/activation of PKC and all the three MAP kinases (ERK, JNK, and p38). Additionally, using potent inhibitors of MAPK, we had previously shown that all these three kinases signal for the production of IL-31 in these HMC-1 cells [18]. These results are similar to those of Liao et al. (2014) [27], who reported that apigenin downregulated the expression of phosphor-ERK and phosphor-JNK in mouse macrophage ANA-1 cells. In contrast to our study, Liao et al. (2014) also showed that apigenin upregulated the expression of phospho-p38 in the ANA-1 cells. This could be explained by the difference in cell type used in the studies, and their activated states thus imply that the action of apigenin can vary with different cell types and activation statue. Nonetheless, we can conclude that one of the mechanisms of action of apigenin in suppressing the production of the IL-31 cytokine is by preventing the activation of MAPK cascade signaling pathways.

It is worthy to note that the effects of apigenin in suppressing IL-31 in HMC-1 cells are not only mediated by MAPK pathways, as NF-κB potent inhibitors also inhibited the production of IL-31 in our previous study [18]. NF-κB/p65 complex is an important transcription factor that is involved in the regulation of most inflammatory genes in cellular processes [28]. In its resting state, its inhibitor (IκB) in the cytoplasm [29] sequesters the NF-κB/p65 complex. Pro-inflammatory cytokines, PI, LPS, growth factors, and antigen receptors can activate IKK complex (IKKβ, IKKα, and IKKγ) through activation of PKC upstream. The activated IKK phosphorylates IκB proteins cause its degradation from the NF-κB/p65 complex. The NF-κB/p65 complex subsequently translocates to the nucleus and leads (either alone or in combination with other transcriptional factors) the transcription and expression of related inflammatory genes [30]. Therefore, we investigated the effects of apigenin in the NF-κB signaling pathways. Preliminary studies also found that the NF-κB/p65 was first significantly expressed 30 min after the HMC-1 were stimulated. Subsequent findings at 30 min after stimulation of the HMC-1 revealed that apigenin suppressed the phosphorylation activation of key signaling molecules in the NF-κB signaling pathway, including IKKβ, IκB, and finally p65. These results implied that the regulatory mechanisms of apigenin in PI-stimulated IL-31 production also involve the modulation of the NF-κB/p65 signaling pathway in HMC-1 cells. It should be noted that these findings are similar to those of other studies, which reported that apigenin modulated the phosphorylation of NF-κB’s p65 subunit via indirect inhibition of the IKKβ kinase activation [31,32,33]. Our results demonstrate, further, the anti-inflammatory property of apigenin and suggest the functional diversity of apigenin in inhibiting inflammatory pathways leading to the production of IL-31.

Another objective of our study was to investigate whether the effects of apigenin on IL-31 expression and production seen in vitro could be translated to an in vivo setting. Several reports have published that flavonoids, especially those closely related in structure to apigenin, like luteolin and quercetin, have anti-itch and anti-allergic properties [34,35]. Since IL-31 is closely linked to the itch in atopic dermatitis, this pushed us to investigate the effects of apigenin in alleviating itch in an atopic dermatitis itch model and to investigate the expression levels of IL-31 in the skin of the mice. Interestingly, in our atopic dermatitis model, the administration of apigenin at a high dose significantly alleviated itch in the mice. Further studies on the mice skin tissues revealed that apigenin also prevented the infiltration of mast cells and their degranulation as evidenced by the decrease in expression of tryptase in the mice skin. Also, the administration of apigenin in the itch model prevented the mRNA and protein expression of IL-31 in the mice skin. These results confirmed our in vitro results on the potential effects of apigenin in inhibiting IL-31 production. In addition, previous reports have shown that apigenin can also prevent histamine release in an in vitro study and IgG levels an in vivo study [36,37]. Our results together with these reports further demonstrate that apigenin can be further evaluated for the treatment of histamine-dependent and histamine-independent itch.

In conclusion, the current results suggest that apigenin has the potential to prevent inflammatory diseases, including alleviating compound 48/80-induced atopic dermatitis itch, through its modulation of IL-31 cytokine. These findings may be applicable in the development of atopic dermatitis itch-reducing or preventing therapies. Long-term and clinical studies are required for the confirmation of the anti-itch effects of apigenin.

## 4. Materials and Methods

### 4.1. Cell Culture and Treatment with Apigenin

Iscove’s modified Dulbecco’s medium (Gibco, Grand Island, NY, USA) supplemented with 10% heat-inactivated fetal bovine serum (Gibco) and 1% penicillin/streptomycin antibiotics (Invitrogen, Carlsbad, CA, USA) was used as culture media for culturing HMC-1 in a humidified incubator at a temperature of 37 °C and CO_2_ level of 5%. The cells (5 × 10^5^ cells/mL) were seeded in sterile 6-well dishes for 16 h and treated with or without apigenin at indicated concentrations for 1 h, after which the cells were stimulated with 50 nM of phorbol-12-myristate 13-acetate and 1 µM of calcium ionophore A23187 (PI) for indicated times. Apigenin with purity of 98% (ChemFaces, Wuhan, China) was dissolved in dimethyl sulfoxide (Sigma-Aldrich, St. Louis, MO, USA) and stored at −20 °C before application. The final concentration of dimethyl sulfoxide in the cell culture media was below the toxicity level of *p* < 0.01%.

For cell viability assay, WST assay was employed to determine cell viability. Cells were pre-treated with various concentrations of apigenin (0, 5, 10, 20, and 30 μM) for 24 h and 0.01 mL of EZ-Cytox reagent (Dogenbio, Seoul, Korea) was added before further incubation for 4 h. After incubation, the absorbance was measured at 540 nm with a microplate reader (Tecan, Männedorf, Switzerland). The absorbance correlates with cell viability.

### 4.2. Animal Care

Male ICR mice five weeks of age with an average weight of 21 ± 1 g were obtained from Orient Bio Inc. (Iksan, Korea). The mice were housed in an air-conditioned room with temperature 22 ± 2 °C, humidity 50–60%, and 12/12 h light–dark cycle. The mice were given a commercial-standard laboratory diet and water at will. All procedures performed complied with the guiding principles for animal care and use committee of Jeonju University Institutional Animal Care and Used Committee guidelines (Approved No. JJU-IACUC-2018-4). Animals were adapted to the laboratory environment for one week prior to experimentation. The number of mice in each experimental group was five.

### 4.3. Atopic Dermatitis Itch Model and Evaluation of Scratching Behavior

Atopic dermatitis itch model was induced as previously described by Orito et al. (2004) [38] with modification. In brief, mice were randomly divided into five groups and orally administered with 75 and 150 mg/kg of apigenin 1 h before the induction of itch. The negative control group and the itch model group were equally administered with 2% gelatin (the vehicle used for apigenin dissolution). To induce itch in the atopic dermatitis itch model and apigenin-treated groups, 50 μg of compound 48/80 was injected into the dorsal dermis of the left shoulder of the mice. Mouse behavior was videotaped with micro-cameras (ONCCTV, Seoul, Korea) for 30 min to check for itching symptoms of atopic dermatitis. Thereafter, the number of times that each mouse in various groups scratched their necks was counted. The Scratching behavior was recorded immediately after injection of compound 48/80.

### 4.4. Measurement of Cytokine Production

HMC-1 cells (5 × 10^5^ cells) were cultured in a sterile 24-well plate. The cells were pre-treated with or without apigenin (10, 20, and 30 µM) for 1 h at 37 °C and then stimulated with PI for 12 h. The supernatants were assayed for IL-31 using an IL-31-specific ELISA kit (Biolegend, San Diego, CA, USA) following the manufacturer’s instructions. Samples were assayed in triplicate.

### 4.5. Protein Extraction

Protein from HMC-1 cells and tissue samples were extracted using a radio-immunoprecipitation assay buffer (RIPA buffer) (Thermo Scientific, Rockford, IL, USA) in accordance with the kit’s enclosed protocol. Protease and phosphatase inhibitors were added to the buffers just before use.

For protein extraction from HMC-1 cells, the cells (5 × 10^5^ cells/mL) were cultured in sterile 6-well dishes and pre-treated with apigenin (10 and 30 µM) for 1 h, after which the cells were stimulated with PI for 30 min or 8 h. The cells were washed with ice-cold PBS and centrifuged at 2500× *g* for 5 min at 4 °C. The supernatant was discarded and the cell pellets were suspended in 0.1 mL of ice-cold RIPA buffer. The tubes were vortexed and incubated on ice for 15 min with gentle shaking. After incubation, the tubes were centrifuged at 14,000× *g* for 15 min at 4 °C to pellet cell debris and the supernatant (protein lysates) transferred into new tubes and stored at −80 °C for subsequent use.

For protein extraction from tissue samples, at the end of the scratching behavior experiment described above, the dorsal dermis at the site of the compound 48/80 injection were removed and 0.2 g of the tissue from each mouse was grounded in liquid nitrogen and placed in 0.2 mL of the RIPA buffer. The tissues were then incubated on ice for 10 min before centrifugation at 19,000× *g* for 10 min at 4 °C. The supernatant was transferred to separate tubes, snap-frozen in liquid nitrogen, and stored at −80 °C for subsequent use.

### 4.6. Western Blotting

Forty μg proteins and 60 μg of protein lysates from HMC-1 and mice tissues, respectively, were loaded onto either 7, 10, or 12% mini-protean TGX precast gels (BIO-RAD, Hercules, CA, USA) and allowed to run at 100 V for 1.5 h. The blots were transferred to Immobilon-P polyvinylidene fluoride membrane (Millipore, Massachusetts, MA, USA) for 1 h at 100 V. The membranes were then blocked with 5% BSA-TBS (*w*/*v*) for 1 h at room temperature and incubated overnight at 4 °C with either antibodies for IL-31, tryptase (Abcam, Cambridge, UK), PKC, phosphor-PKC, PKC, ERK, phosphor-ERK, JNK, phosphor-JNK, p38, phospho-p38, IKKβ, Phosphor-IKKβ, IKBα, phosphor-IKBα, NF-κB, or phosphor-NF-κB (Santa Cruz Biotechnology, Dallas TX, USA). To ensure equal protein loading, the membranes were stripped and reprobed with β-actin (Biosciences, San Diego, CA, USA) or tubulin antibodies (Santa Cruz Biotechnology). Blots were incubated in respective secondary antibodies (Santa Cruz Biotechnology, CA, USA) in 5% milk-TBS for 2 h at room temperature and then visualized on an ultraviolet detection Imaging System using an enhanced chemiluminescence detection kit (Amersham Biosciences, Piscataway, NJ, USA). Band intensity was measured using the Image J gel analysis software.

### 4.7. RNA Extraction and Reverse Transcription (RT)-PCR Analysis of IL-31 mRNA Levels

For HMC-1 RNA extractions, the cells were cultured in the presence or absence of apigenin (10 and 30 µM) and stimulated with PI for 3 h. The cells were then harvested and placed on ice for RNA extraction. The RNA was isolated and purified using Ribospin II extraction kit (GeneAll Biotechnology, Seoul, Korea) in accordance with the manufacturer’s specifications and stored at −20 °C. For tissue RNA extraction, 0.2 g of tissue from each mouse was ground to fine powder with liquid nitrogen in a pre-chilled mortar and pestle. The RNA was then isolated and purified using Ribospin II extraction kit (GeneAll Biotechnology) and stored at −20 °C. One microgram of purified RNA from both HMC-1 and tissue samples was reverse-transcribed to cDNA using a PrimeScript™ RT Master Mix (Takara Bio Inc., Nojihigashi, Japan) following the manufacturer’s protocol with a T100^TM^ Bio-Rad Thermal Cycler set at 42 °C for 1 h. cDNA was amplified with Stratagene Mx3000P (Agilent) using the SYBR Premix Ex Taq™ (Takara Bio Inc, Nojihigashi, Japan) according to manufacturer instructions. The standard cycle settings were as follows: 95 °C for 5 min, 40× (95 °C for 30 s, 62 °C for 30 s) followed by a dissociation curve ramping from 95 °C to 55 °C and back to 95 °C. For IL-31, the following primers were used: Sense, 5′tgtgccaacagacacccatg3′ and antisense, 5′tgttgggctccagaggtcaa3′. GAPDH was selected as a reference gene with the following primers: Sense, 5′cactcctccacctttgacgc3′ and antisense, 5′tccaccaccctgttgctgta3′. All samples were run in triplicate and the expression levels were normalized with GAPDH using the 2^−ΔΔCt^ comparative method.

### 4.8. Histopathological and Immunohistochemistry Examination

At the end of the itching experiment, the dorsal skin tissues at the site of compound 48/80 injections were excised and fixed with 4% paraformaldehyde for 24 h. The tissue sections were dehydrated in graded alcohol concentrations, cleared in two changes of xylene, and embedded and blocked in paraffin. Four micrograms of tissue sections were obtained and stained with hematoxylin and eosin (H&E) and toluidine blue for mast cell analysis. For immunochemical analysis, antigen retrieval using citrate buffer at pH 6.0 and tissue permeabilization with 5% saponin were performed on the 4 μm-thin tissue sections. The tissue sections were incubated in normal horse serum (2.5%) for 20 min before subsequent incubation with mouse IL-31 and tryptase antibodies (Abcam) for 16 h at 4 °C. After incubation with primary antibodies, the sections were further incubated for 30 min with ImmPRESS^TM^ HRP reagent conjugated with horse anti-mouse IgG. (Vector laboratory, Burlingame, CA, USA). The tissue sections were then incubated in AEC peroxidase substrate (Vector laboratory) for 10 min, mounted with an aqueous-based mounting medium (ImmunoHistoMount^TM^, Sigma-Aldrich) and visualized under the light microscope.

### 4.9. Statistical Analysis

All data from this study were analyzed with the one-way ANOVA–Duncan’s tests by using IBM Statistical Package for Social Sciences (SPSS) version 20.0 statistical software (IBM, Armonk, NY, USA). The differences between the groups were said to be statistically significant at *p* ≤ 0.05.
